# Two new species of *Ancystrocerus* Raffray from the Oriental region (Coleoptera, Staphylinidae, Pselaphinae)

**DOI:** 10.3897/zookeys.958.54196

**Published:** 2020-08-11

**Authors:** Zi-Wei Yin

**Affiliations:** 1 Laboratory of Systematic Entomology, College of Life Sciences, Shanghai Normal University, 100 Guilin Road, Shanghai 200234, China Shanghai Normal University Shanghai China

**Keywords:** ant-loving beetle, biodiversity, China, Philippines, taxonomy

## Abstract

Two new species of the genus *Ancystrocerus* Raffray, *A. lueliangi***sp. nov.** (China: Yunnan) and *A. philippinus***sp. nov.** (Philippines: Mindanao), are described, illustrated, and distinguished from related congeners.

## Introduction

The genus *Ancystrocerus* Raffray of the pselaphine tribe Tmesiphorini currently comprises ten species distributed in Indonesia (4 species), Singapore (3 species), Malaysia (2 species, 1 shared with Singapore), India (1 species), and China (1 species) (see Yin et al. 2015 for a historical review, and references therein; Newton 2020). Members of *Ancystrocerus* differ from those of all other Oriental genera of the tribe by the elongate habitus and appendages, notably long and slender maxillary palpi, and often modified antennomeres 9 and 10 in the male. The only recently described species, *A. chinensis* Yin, Wang & Li from Hainan Island, southern China, represents the northernmost record of the genus (Yin et al. 2015). The biology of the genus is largely unknown, three species (*A. rugicollis* Raffray, *A. punctatus* Raffray, *A. longicornis* Raffray) were collected by sifting, one (*A. pallidus* Raffray) was taken under bark of a rotten tree (Raffray 1895), and *A. chinensis* was found in decomposing logs inhabited by termites (Yin et al. 2015).

In the present paper, two new species occurring in China (Yunnan) and the Philippines (Mindanao) are described, one of which was sifted from a leaf litter sample in a broad-leaved forest.

## Materials and methods

The type material of the new species described in this paper is deposited in the Insect Collection of Shanghai Normal University, Shanghai, China (**SNUC**). The text of the specimen label is quoted verbatim in quotation marks (‘’).

Male genital parts (tergite and sternite VIII, and aedeagus) were dissected, and are preserved in Euparal on a plastic slide pinned beneath the specimen. The habitus images were taken using a Canon 5D Mark III camera in conjunction with a Canon MP-E 65 mm f/2.8 1–5× Macro Lens, and a Canon MT-24EX Macro Twin Lite Flash was used as the light source. Images of the external characters were taken using a Leica DMC5400 color CMOS camera in conjunction with a Leica M205 C stereomicroscope. Images of the aedeagi were produced using a Canon G9 camera mounted to an Olympus CX31 microscope under transmitted light. Zerene Stacker (version 1.04) was used for image stacking. Line drawings were made using Adobe Illustrator CC 2018. All images were optimized and grouped into plates using Adobe Photoshop CC 2018.

The abdominal tergites and sternites are numbered following Chandler (2001) in Arabic (starting from the first visible segment) and Roman (reflecting true morphological position) numerals, e.g., tergite 1 (IV), or sternite 1 (III).

## Taxonomy

### Ancystrocerus lueliangi sp. nov.

Taxon classificationAnimaliaColeopteraStaphylinidae

0224CE5A-2935-5949-B4DA-BBD3BF15C67B

http://zoobank.org/916A4D1B-B395-4318-BAEB-F73205C4C10A

Fig. 1

#### Type material.

***Holotype*: China**: ♂, ‘China: Yunnan, Yingjiang Co. (盈江县), Tongbiguan N. R. (铜壁关自然保护区), 24.6136444N, 97.5851155E, 1255 m, 28.viii.2019, Liang Lü leg.’ (SNUC).

#### Diagnosis.

**Male.** Length 2.4 mm (combined length of head, pronotum, elytra and abdomen). Head and pronotum roughly punctate. Antennomeres 4 and 5 subequal in length, 9 and 10 expanded laterally, apex of antennomeres 9 and base of antennomeres 10 obliquely constricted, each with one bunch of bristles. Pronotum with small, conical discal spine. Tergites 1 and 2 (IV and V) with median carina extending through entire tergal length. Aedeagus relatively more slender, median lobe symmetrical in dorso-ventral view; endophallus with single long sclerite and two pairs of short sclerites; parameres each elongate and with two long apical setae.

#### Description.

**Figure 1.**
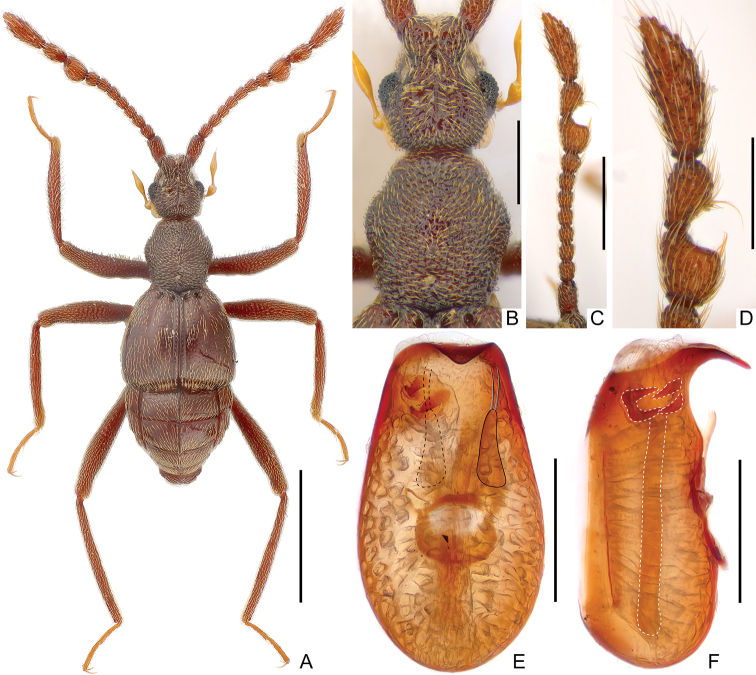
*Ancystrocerus lueliangi* sp. nov., male **A** habitus **B** head dorsum and pronotum **C** right antenna **D** antennal club of right antenna **E, F** aedeagus in ventral (**E**) and lateral (**F**) view. Scale bars: 1.0 mm (**A**); 0.3 mm (**B, D**); 0.5 mm (**C**); 0.2 mm (**E, F**).

**Male** (Fig. 1A). ***Length*** 2.4 mm. ***Head*** (Fig. 1B) slightly longer than wide, length from anterior margin of clypeus to posterior margin of vertex (excluding occipital construction) 0.51 mm, width across eyes 0.43 mm; dorsal surface roughly punctate; postocular margins with dense tufted hairs; eyes prominent, each composed of about 35 facets. ***Antennae*** (Fig. 1C) elongate, with clubs (Fig. 1D) formed by apical three antennomeres; scapes large, antennomeres 2 smaller than scapes, antennomeres 3–8 each subquadrate, 8 slightly transverse and larger than each of antennomeres 2–7, antennomeres 9–10 modified, antennomeres 11 largest, elongate, widest at middle. ***Pronotum*** (Fig. 1B) slightly longer than wide, length along midline 0.56 mm, maximum width 0.52 mm, roughly punctate, disc with small, acute denticle at middle. ***Elytra*** much wider than long, length along suture 0.66 mm, maximum width 0.87 mm; shallow and broad discal striae extending posteriorly to past half of elytral length. ***Legs*** simple, elongate. ***Abdomen*** wider than long, length along midline 0.66 mm, maximum width 0.87 mm. ***Tergite*** 1 (IV) approximately as long as tergite 2 (V), both tergites with entire and distinct median carina. Length of aedeagus (Fig. 1E, F) 0.47 mm, elongate, well sclerotized; median lobe curved ventrally at apex in lateral view; endophallus composed of one elongate sclerite at middle and two short and strongly sclerotized sclerites at apex; parameres elongate and flattened, each with two long setae at apex.

**Female.** Unknown.

#### Comparative notes.

Males of the new species can be readily separated from *A. chinensis* and all other congeners by the roughly punctate head and pronotum, and the different shape of antennomeres 9–10 and structure of the aedeagus. *Ancystrocerus chinensis* from Hainan has finely punctate head and pronotum, the antennomeres 9 and 10 more elongate, and aedeagus much more robust and with a large projection at the apex of the median lobe.

#### Distribution.

China: Yunnan.

#### Biology.

The single male was sifted from a leaf litter sample in a shady broad-leaved forest.

#### Etymology.

The new species is named after Liang Lü, collector of the holotype.

### Ancystrocerus philippinus sp. nov.

Taxon classificationAnimaliaColeopteraStaphylinidae

5AB3AFE9-C5E5-5F80-9959-95E1E583BDF5

http://zoobank.org/D6BC17D4-ACC9-4F59-B545-C4A7D2F63C37

Fig. 2

#### Type material.

***Holotype*: Philippines**: ♂, ‘Philippines: Alamada, North Corabato, Mindanao, i.2019, local collector’ (SNUC).

#### Diagnosis.

**Male.** Length 2.6 mm (combined length of head, pronotum, elytra and abdomen). Head and pronotum finely punctate. Antennomeres 4 longer than 5, antennomeres 9 expanded and projected laterally, 10 hardly so, lateral margin of antennomeres 9 and 10 with one bunch of bristles. Pronotum with small, conical discal spine. Tergite 1 (IV) with median carina extending through entire tergal length, tergite 2 lacking such carina. Aedeagus relatively stout, median lobe symmetrical in dorso-ventral view; endophallus with several short and two elongate sclerites; parameres each elongate and with two long apical setae.

#### Description.

**Figure 2.**
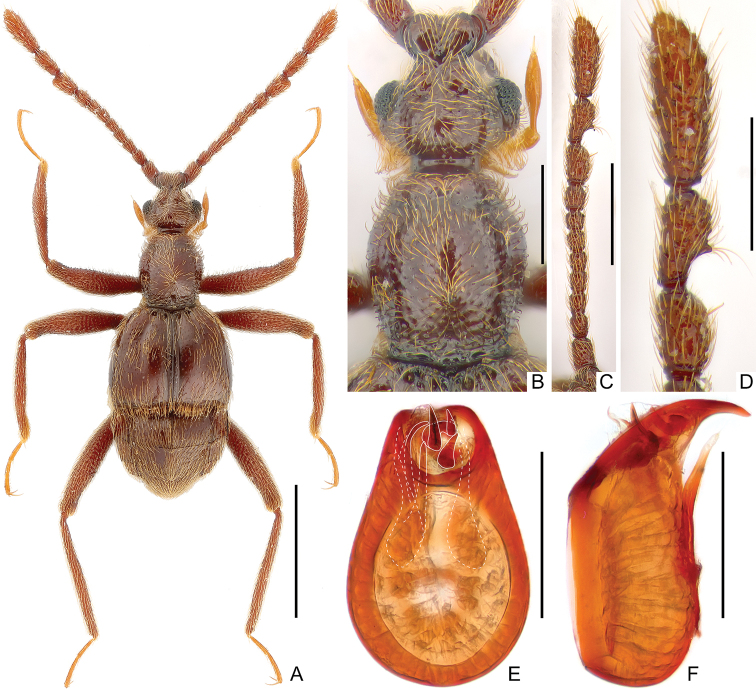
*Ancystrocerus philippinus* sp. nov., male **A** habitus **B** head dorsum and pronotum **C** right antenna **D** antennal club of right antenna **E, F** aedeagus in dorsal (**E**) and lateral (**F**) view. Scale bars: 1.0 mm (**A**); 0.3 mm (**B, D**); 0.5 mm (**C**); 0.2 mm (**E, F**).

**Male** (Fig. 2A). ***Length*** 2.6 mm. ***Head*** (Fig. 2B) as long as wide, length from anterior margin of clypeus to posterior margin of vertex (excluding occipital construction) 0.46 mm, width across eyes 0.46 mm; dorsal surface finely punctate; postocular margins with rather dense tufts of hairs; eyes prominent, each composed of about 55 facets. ***Antennae*** (Fig. 2C) elongate, with clubs (Fig. 2D) formed by apical three antennomeres; scapes large, antennomeres 2 smaller than scapes, antennomeres 3–8 each subquadrate, 8 as long as wide and larger than each of antennomers 2–7, antennomeres 9–10 modified, antennomeres 11 largest, elongate, widest at 1/3. ***Pronotum*** (Fig. 2B) slightly longer than wide, length along midline 0.58 mm, maximum width 0.49 mm, finely punctate, disc with small, acute spine at middle. ***Elytra*** much wider than long, length along suture 0.74 mm, maximum width 0.94 mm; shallow broad discal striae extending posteriorly to less than half elytral length. Legs simple, elongate. ***Abdomen*** wider than long, length along midline 0.78 mm, maximum width 0.87 mm. ***Tergite*** 1 (IV) approximately as long as tergite 2 (V), tergite 1 with entire and distinct median carina, which is absent on tergite 2. Length of aedeagus (Fig. 2E, F) 0.35 mm, relatively stout, well sclerotized; median lobe curved ventrally at apex in lateral view; endophallus composed of three thick and short sclerites at apex and two elongate ones on the left; parameres elongate and flattened, each with two long setae at apex.

**Female.** Unknown.

#### Comparative notes.

Males of the new species can be readily separated from all congeners by the presence of very dense hairs along the postocular margins, the unique shape of antennomeres 9 and 10, and the structure of the aedeagal endophallus. The elongate antennomeres 9 and 10 of *A. philippinus* are somewhat similar to those of *A. chinensis*, however, the antennomeres 9 are much less expanded in the new species and the aedeagus is totally different in form and structure. *Ancystrocerus philippinus* further differs by tergite 1 with a complete median carina, which in *A. chinensis* is short and extending posteriorly only to less than half of tergal length.

The antennomeres 10 of *A. irregularis* Raffray, *A. sumatrensis* Raffray, *A. rugicollis* Raffray, and *A. punctatus* Raffray all bear one bunch of bristles on the lateral surface. In addition to the form of the aedeagal median lobe, the unique structure of the endophallus, and the different proportions of antennomeres 9–11, *A. philippinus* can be readily separated from the former three species by the presence of a thin bunch of bristles also on antennomeres 9, and from *A. punctatus* by the smooth mesal margin of antennomeres 10 (strongly protuberant in *A. punctatus*).

#### Distribution.

Philippines: Mindanao.

#### Biology.

Unknown.

#### Etymology.

The specific epithet refers to the Philippines, where the type locality of the new species is located.

## Supplementary Material

XML Treatment for Ancystrocerus lueliangi

XML Treatment for Ancystrocerus philippinus
